# Human Papillomavirus Vaccination Coverage Among Adolescents, 2007–2013, and Postlicensure Vaccine Safety Monitoring, 2006–2014 — United States

**Published:** 2014-07-25

**Authors:** Shannon Stokley, Jenny Jeyarajah, David Yankey, Maria Cano, Julianne Gee, Jill Roark, C. Robinette Curtis, Lauri Markowitz

**Affiliations:** 1 Immunization Services Division, National Center for Immunization and Respiratory Diseases, CDC; 2 Immunization Safety Office, National Center for Emerging, Zoonotic, and Infectious Diseases, CDC; 3 Health Communications Science Office, National Center for Immunization and Respiratory Diseases, CDC; 4 Division of Sexually Transmitted Diseases, National Center for HIV/AIDS, Viral Hepatitis, STD, and TB Prevention, CDC

Since mid-2006, a licensed human papillomavirus (HPV) vaccine has been available and recommended by the Advisory Committee on Immunization Practices (ACIP) for routine vaccination of adolescent girls at ages 11 or 12 years (1). Two vaccines that protect against HPV infection are currently available in the United States. Both the quadrivalent (HPV4) and bivalent (HPV2) vaccines protect against HPV types 16 and 18, which cause 70% of cervical cancers; HPV4 also protects against HPV types 6 and 11, which cause 90% of genital warts (1,2). In 2011, the ACIP also recommended HPV4 for the routine vaccination of adolescent boys at ages 11 or 12 years (3). HPV vaccines can be safely co-administered with other routinely recommended vaccines, and ACIP recommends administration of all age-appropriate vaccines during a single visit (4). To assess progress with HPV vaccination coverage among adolescents aged 13–17 years,* characterize adherence with recommendations for HPV vaccination by the 13th birthday, and describe HPV vaccine adverse reports received postlicensure, CDC analyzed data from the 2007–2013 National Immunization Survey-Teen (NIS-Teen) and national postlicensure vaccine safety data among females and males. Vaccination coverage with ≥1 dose of any HPV vaccine increased significantly from 53.8% (2012) to 57.3% (2013) among adolescent girls and from 20.8% (2012) to 34.6% (2013) among adolescent boys. Receipt of ≥1 dose of HPV among girls by age 13 years increased with each birth cohort; however, missed vaccination opportunities were common. Had HPV vaccine been administered to adolescent girls born in 2000 during health care visits when they received another vaccine, vaccination coverage for ≥1 dose by age 13 years for this cohort could have reached 91.3%. Postlicensure monitoring data continue to indicate that HPV4 is safe. Improving practice patterns so that clinicians use every opportunity to recommend HPV vaccines and address questions from parents can help realize reductions in vaccine-preventable infections and cancers caused by HPV.

## Vaccination Coverage

Since 2006, NIS-Teen has collected vaccination information for adolescents aged 13–17 years in the 50 states, the District of Columbia, and selected areas,† using a random-digit–dialed sample of landline and, starting in 2011, cell phone numbers.§ After a teen’s parent/guardian grants permission to contact their teen’s vaccination provider(s), a questionnaire is mailed to each provider to obtain a vaccination history from medical records.¶ Analysis for this report was limited to adolescent girls and boys with provider-reported vaccination histories.** HPV vaccination coverage represents receipt of any HPV vaccine and does not distinguish between HPV2 or HPV4. NIS-Teen methodology, including weighting procedures, has been described previously (5). Differences in vaccination coverage were evaluated using t-tests and were considered statistically significant if p<0.05.

Vaccination coverage was assessed for each dose of the HPV vaccination series. For girls and boys, respectively, and for each vaccine series dose, HPV vaccination coverage estimates in 2013 were significantly higher compared with 2012 (Table 1).

To evaluate receipt of ≥1 dose of HPV vaccine by age 13 years among adolescent girls, data during 2007–2013 NIS-Teen survey years were combined and analyzed by birth cohort.†† Among girls, receipt of ≥1 dose of HPV by age 13 years has increased an average of 5.9% (95% confidence interval [CI] = 2.8%–9.0%) with each new birth cohort, reaching 46.8% (CI = 41.2%–52.5%) for the 2000 birth cohort (Figure). Missed opportunities to receive the HPV vaccine by age 13 years also were evaluated. A missed opportunity for adolescent girls was defined as a health care encounter occurring on or after the 11th birthday and before the 13th birthday and on or after March 23, 2007 (the publication date of the ACIP HPV4 recommendation for girls), during which the adolescent received at least one vaccine but did not receive the first dose of the HPV vaccine. The percentage of unvaccinated girls at age 13 years with at least one missed opportunity for HPV vaccination ranged from 9.3% (CI = 8.1%–10.8%) for the 1994 cohort to 83.7% (CI = 77.8%–88.2%) for the 2000 cohort (Figure). If all missed opportunities for HPV vaccination had been eliminated for the 2000 birth cohort, vaccination coverage with ≥1 dose of HPV vaccine could have reached 91.3% (CI = 87.9%–93.8%) by age 13 years, a 42.7 percentage-point difference from the actual coverage level.

The percentage of parents reporting that they received a recommendation for the HPV vaccine from their clinician was significantly higher in 2013 compared with 2012 for both parents of girls (64.4% compared with 61.0%) and parents of boys (41.6% compared with 28.0%). More parents of vaccinated teens (girls: 73.7%; boys: 71.7%) reported receiving a recommendation compared with parents of unvaccinated teens (girls: 52.0%; boys: 25.7%).

The 2013 NIS-Teen asked parents who reported they were not likely to vaccinate their teen in the 12 months after interviews or were unsure of their vaccination plans (girls: 23.0% [CI = 21.5%–24.6%]; boys: 37.4% [CI = 35.7%–39.1%]) to identify the main reason why their teen would remain unvaccinated. The top five responses from the parents of girls and parents of boys were the same, differing only in order of frequency (Table 2). More than 30% of the parents of girls and boys cited as their main reason lack of knowledge (girls and boys: both 15.5%) or belief that the vaccine was not needed or necessary (girls: 14.7%; boys: 17.9%). Among parents of boys, 22.8% reported that the main reason was that HPV vaccination had not been recommended; among parents of girls, 13.0% reported that HPV had not been recommended.

## Vaccine Safety

In the United States, postlicensure vaccine safety monitoring and evaluation are conducted independently by federal agencies and vaccine manufacturers. From June 2006 through March 2014, approximately 67 million doses of HPV4 were distributed in the United States, and from October 2009 through March 2014, a total of 719,000 doses of HPV2 were distributed. Overall, HPV4 has accounted for approximately 99% of doses distributed since 2006. Multiple studies have provided evidence supporting HPV vaccine safety (6). During June 2006–March 2014, the Vaccine Adverse Event Reporting System (VAERS)§§ received a total of 25,176 adverse event reports after HPV vaccination in the United States. Among these, HPV4 was cited in 99% of reports (22,867 and 2,196 reports among females and males, respectively); 92.4% of the HPV4 reports were classified as nonserious.¶¶ Since October 2009, when HPV4 was licensed for males, the most commonly reported symptoms among males were similar to those among females, including injection site reactions, dizziness, syncope, nausea, and headache. Overall, reporting of adverse events to VAERS is consistent with prelicensure clinical trial data and consistent with the 2009 published summary of the first 2.5 years of postlicensure reporting to VAERS (7).

### Discussion

After a year of unchanging HPV vaccination coverage among adolescent girls (6), results from the 2013 NIS-Teen show a modest increase in coverage; however, coverage levels remain low. From 2012 to 2013, the percentage of adolescents receiving ≥1 dose of HPV vaccine increased 3.5 percentage points for girls and 13.8 percentage points for boys. A cohort analysis also was performed to evaluate receipt of ≥1 dose of HPV vaccine by age 13 years over time and found an increase since 2007; however, missed vaccination opportunities persist. Had HPV vaccine been administered during health care visits when another vaccine was administered, vaccination coverage for ≥1 dose could have reached 91.3% by age 13 years for adolescent girls born in 2000.

Despite availability of safe and effective HPV vaccines, the main reasons reported for not vaccinating teens against HPV underscore that addressing knowledge gaps among parents as well as increasing clinicians’ HPV vaccination recommendations are critical to protecting teens against HPV-associated cancers and genital warts. In 2013, the percentage of parents who reported receiving a recommendation for the HPV vaccine increased. Nevertheless, it is concerning that approximately one third of parents of girls and over half of parents of boys reported that their child’s clinician had not recommended that their child receive an HPV vaccination. The lack of a clinician recommendation among parents of boys might reflect knowledge limitations among clinicians because the recommendation for routine HPV vaccination for boys has only been in place since December 2011. HPV infections can cause serious, life-threatening cancers among men (3); it is important to continue to educate vaccination providers and parents to ensure that adolescent boys are protected from HPV-associated cancers and genital warts.

The President’s Cancer Panel 2012–2013 report released in February 2014 (available at http://deainfo.nci.nih.gov/advisory/pcp/annualreports/hpv/index.htm) recommended three critical goals that must be achieved to increase HPV vaccination coverage in the United States, including 1) reducing missed clinical opportunities to recommend and administer HPV vaccines; 2) increasing parents’, caregivers’, and adolescents’ acceptance of HPV vaccination; and 3) maximizing access to HPV vaccination services. CDC, in partnerships with state and local immunization programs, is working with health professional organizations to reduce missed opportunities for HPV vaccination and support clinicians’ capacities to give HPV vaccination recommendations consistent with national vaccination recommendations.

What is already known on this topic?

The Advisory Committee on Immunization Practices recommends human papillomavirus (HPV) vaccination for girls and boys at ages 11 or 12 years. The 2012 National Immunization Survey-Teen indicated only 53.8% of girls and 20.8% of boys aged 13–17 years had received ≥1 dose of HPV vaccine.

What is added by this report?

Vaccination coverage significantly increased in 2013; 57.3% of girls and 34.6% of boys received ≥1 dose of HPV vaccine. The percentage of parents reporting that they received a clinician recommendation for the HPV vaccine was significantly higher in 2013 compared with 2012 for both parents of girls (64.4% versus 61.0%) and parents of boys (41.6% versus 28.0%). Analysis of provider records showed that if HPV vaccine had been administered at health care encounters when other recommended vaccines were administered, ≥1 HPV vaccination coverage by age 13 years for the most recent birth cohort of girls could have been as high as 91%. National safety monitoring data continue to indicate that the HPV vaccine is safe.

What are the implications for public health practice?

Despite the availability of safe and effective HPV vaccines, many adolescents have not been vaccinated. Vaccination coverage of adolescent girls by age 13 years increased across seven birth cohorts but missed vaccination opportunities persist. Improving practice patterns so that clinicians and their staff members use every opportunity to recommend HPV vaccines for boys and girls and address questions from parents is necessary to reduce vaccine-preventable HPV infections and cancers caused by HPV.

To address gaps in clinician knowledge and communication skills, several resources have been developed by CDC including a dedicated website for health care professionals on HPV vaccine resources (http://www.cdc.gov/vaccines/youarethekey), a tip sheet for talking about the HPV vaccine with parents, and continuing education programs for pediatricians and family physicians regarding the clinical impact of persistent HPV infection and the importance of vaccinating adolescents at ages 11–12 years. To improve public acceptance of HPV vaccination, CDC continues to use research data to create an evidence-based communication campaign to reach the target audiences. Although it is still too early to evaluate the impact of activities implemented since publication of the 2012 NIS-Teen results (6,8), which documented that HPV vaccination coverage rates among girls did not increase compared with 2011, results from the 2013 NIS-Teen indicate that initial progress has been made.

The findings in this report are subject to at least four limitations. First, the cell phone household response rate was only 23.3%, and the landline household response rate was only 51.1%. Sampling weights were designed to minimize nonresponse and noncoverage bias (from exclusion of households without landline telephones), but some bias might remain in weighted estimates. Second, vaccination histories reported by providers might be incomplete, which would contribute to underestimation of vaccination coverage. Third, evaluation of missed opportunities only included health care encounters in which a vaccination was administered, and thus estimates of potential coverage would be underestimated if there were additional health care encounters in which a vaccination could have been administered. Finally, VAERS is a passive reporting system that accepts reports from anyone, including health care providers, patients, or family members. VAERS cannot determine cause and effect; a report of an adverse event to VAERS does not mean that a vaccine caused the event. Underreporting might occur, and serious medical events are more likely to be reported than minor ones.

The cohort analysis presented in this report combines data from subjects over multiple survey years; the denominator for the 2000 cohort might not be the same as the denominator for females aged 13 years included in the 2013 NIS-Teen data (9). In addition, the cohort analysis focuses on coverage by age 13 years, whereas 2013 NIS-Teen analyses for females aged 13 years could reflect doses that were received by girls after their 13th birthday and before interview dates. As a result, the cohort estimate is lower than that reported for females aged 13 years (9).

Progress with HPV vaccination is occurring, but at a slow pace. In 2013, only 57.3% of girls and 34.6% of boys had initiated the HPV vaccine series. CDC will continue its efforts to partner with state and local immunization programs, professional organizations, cancer organizations, and other stakeholders to educate parents and clinicians. Collaborative efforts remain critical to promoting HPV vaccination so that the nation’s adolescents are protected against vaccine-preventable, HPV-associated cancers.

## Figures and Tables

**FIGURE:**
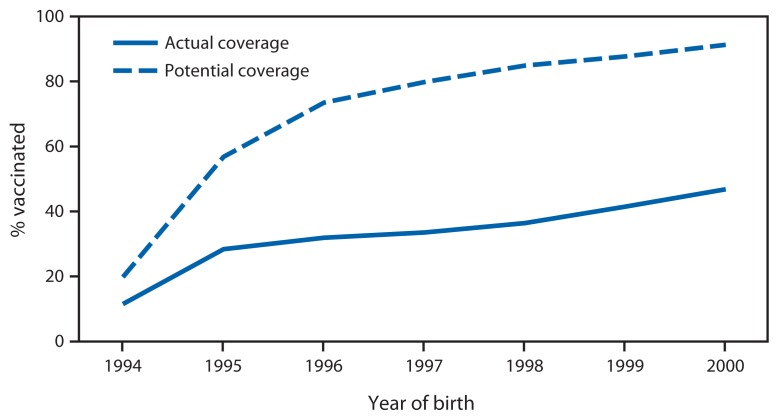
Actual and potentially achievable vaccination coverage with ≥1 dose of human papillomavirus (HPV) vaccine if missed vaccination opportunities had been eliminated among girls by age 13 years,* by birth cohort (1994–2000) — National Immunization Survey-Teen, United States, 2007–2013 combined * Missed opportunity was defined as a health care encounter occurring on or after a girl’s 11th birthday and before her 13th birthday, and on or after March 23, 2007, during which a girl received at least one vaccination, but not the first dose of the HPV vaccine series.

**TABLE 1:** Estimated human papillomavirus vaccination* coverage among adolescent boys and girls aged 13–17 years — National Immunization Survey-Teen, United States, 2007–2013

	Survey year†													
	
	2007		2008		2009		2010		2011		2012		2013	
							
Sex/Doses	%	(95% CI)	%	(95% CI)	%	(95% CI)	%	(95% CI)	%	(95% CI)	%	(95% CI)	%	(95% CI)
Adolescent girls														
≥1 dose	25.1	(22.3–28.1)	37.2	(35.2–39.3)§	44.3	(42.4–46.1)§	48.7	(46.9–50.5)§	53.0	(51.4–54.7)§	53.8	(52.0–55.7)	57.3	(55.4–59.2)§
≥2 dose	16.9	(14.6–19.6)	28.3	(26.4–30.3)§	35.8	(34.1–37.6)§	40.7	(38.9–42.5)§	43.9	(42.3–45.6)§	43.4	(41.5–45.2)	47.7	(45.7–49.6)§
≥3 dose	5.9	(4.4–7.8)	17.9	(16.3–19.6)§	26.7	(25.2–28.3)§	32.0	(30.3–33.6)§	34.8	(33.2–36.4)§	33.4	(31.7–35.2)	37.6	(35.7–39.6)§
Adolescent boys														
≥1 dose	—	—	—	—	—	—	—	—	8.3	(7.4–9.3)	20.8	(19.4–22.4)§	34.6	(32.7–36.5)§
≥2 dose	—	—	—	—	—	—	—	—	3.8	(3.2–4.5)	12.7	(11.5–14.0)§	23.5	(21.8–25.3)§
≥3 dose	—	—	—	—	—	—	—	—	1.3	(1.0–1.7)	6.8	(5.9–7.8)§	13.9	(12.5–15.3)§

**Abbreviation:** CI = confidence interval.

* Human papillomavirus vaccine, either quadrivalent or bivalent.

† The number of adolescent girls with provider reported vaccination histories for each survey year were as follows: 2007, 1,440; 2008, 8,607; 2009, 9,621; 2010, 9,220; 2011, 11,236; 2012, 9,058; and 2013, 8,710. The number of adolescent boys with provider reported vaccination histories for each survey year were as follows: 2011, 12,328; 2012, 10,141; and 2013, 9,554.

§ Statistically significant difference (p<0.05) compared with the previous year’s estimate.

**TABLE 2:** Top five reasons for not vaccinating adolescents with human papillomavirus (HPV) vaccine* — National Immunization Survey-Teen, United States, 2013

Parents of girls			Parents of boys		
	
Reason	%	(95% CI)	Reason	%	(95% CI)
Lack of knowledge	15.5	(13.0–18.5)	Not recommended	22.8	(20.6–25.0)
Not needed or necessary	14.7	(12.5–17.3)	Not needed or necessary	17.9	(15.9–20.1)
Safety concern/Side effects	14.2	(11.8–16.8)	Lack of knowledge	15.5	(13.7–17.6)
Not recommended	13.0	(10.8–15.5)	Not sexually active	7.7	(6.4–9.2)
Not sexually active	11.3	(9.1–13.9)	Safety concern/Side effects	6.9	(5.6–8.5)

**Abbreviation:** CI = confidence interval.

* Analysis limited to parents reporting that they were not likely to seek HPV vaccination for their teen in the next 12 months or were unsure of their HPV vaccination plans.

## Footnotes

* For each survey year, eligible participants were born during the following periods: 2007, October 1989–February 1995; 2008, January 1990–February 1996; 2009, January 1991–February 1997; 2010, January 1992–February 1998; 2011, January 1993–February 1999; 2012, January 1994–February 2000; and 2013, January 1995–February 2001.

† Six areas that received federal Section 317 immunization grants were sampled separately: District of Columbia; Chicago, Illinois; New York, New York; Philadelphia County, Pennsylvania; Bexar County, Texas; and Houston, Texas.

§ All identified cell phone households were eligible for interview. Sampling weights were adjusted to correct for dual-frame (landline and cell phone) sampling, nonresponse, noncoverage, and overlapping samples of mixed telephone users. A description of NIS-Teen dual-frame survey methodology and its effect on reported vaccination estimates is available at http://www.cdc.gov/vaccines/stats-surv/nis/dual-frame-sampling-082812.htm.

¶ In 2013, the Council of American Survey Research Organizations (CASRO) landline response rate was 51.1%. A total of 6,039 adolescents with vaccination provider–reported vaccination records were included, representing 59.5% of all adolescents from the landline sample with completed household interviews. The cell phone sample CASRO response rate was 23.3%. A total of 12,225 adolescents with vaccination provider–reported vaccination records are included, representing 54.5% of all adolescents from the cell phone sample with completed household interviews. The CASRO response rate is the product of three other rates, 1) the resolution rate, which is the proportion of telephone numbers that can be identified as either for a business or residence; 2) the screening rate, which is the proportion of qualified households that complete the screening process; and 3) the cooperation rate, which is the proportion of contracted eligible households for which a completed interview is obtained. CASRO response rates for survey years 2007–2012 are available at http://www.cdc.gov/nchs/nis/data_files_teen.htm.

** The number of adolescent girls with provider-reported vaccination histories for each survey year were as follows: 2007, 1,440; 2008, 8,607; 2009, 9,621; 2010, 9,220; 2011, 11,236; 2012, 9,058; and 2013, 8,710. The number of adolescent boys with provider-reported vaccination histories for each survey year were as follows: 2011, 12,328; 2012, 10,141; and 2013, 9,554.

†† This analysis was not performed for adolescent boys because the majority of the boys included in the NIS-Teen sample were unable to receive the vaccine by age 13 years because of their birthdates relative to the 2011 recommendation to vaccinate males.

§§ Additional information about the Vaccine Adverse Event Reporting System (VAERS) is available at http://www.cdc.gov/vaccinesafety/activities/vaers.html.

¶¶ Serious reports are those in which one or more of the following were noted in the report: death, life-threatening illness, hospitalization, prolongation of an existing hospitalization, or permanent disability.
